# Rewiring the Brain Through the Gut: Insights into Microbiota–Nervous System Interactions

**DOI:** 10.3390/cimb47070489

**Published:** 2025-06-26

**Authors:** Ilinca Savulescu-Fiedler, Serban-Nicolae Benea, Constantin Căruntu, Andreea-Simona Nancoff, Corina Homentcovschi, Sandica Bucurica

**Affiliations:** 1 Department of Internal Medicine, “Carol Davila” University of Medicine and Pharmacy, 050474 Bucharest, Romania; ilinca.savulescu@umfcd.ro (I.S.-F.); corina.homentcovschi@umfcd.ro (C.H.); 2 Department of Internal Medicine, Coltea Clinical Hospital, 030167 Bucharest, Romania; 3 Department of Infectious Diseases, “Carol Davila” University of Medicine and Pharmacy, 050474 Bucharest, Romania; 4 National Institute for Infectious Diseases “Prof. Dr. Matei Bals”, 021105 Bucharest, Romania; 5 Department of Physiology, “Carol Davila” University of Medicine and Pharmacy, 050474 Bucharest, Romania; 6 Department of Dermatology, “Prof. N.C. Paulescu” National Institute of Diabetes, Nutrition and Metabolic Diseases, 011233 Bucharest, Romania; 7 Department of Gastroenterology, Bucharest Emergency Clinical Hospital, 014461 Bucharest, Romania; andreea-simona.nancoff@rez.umfcd.ro; 8 Department of Gastroenterology, “Carol Davila” University of Medicine and Pharmacy, 050474 Bucharest, Romania; sandica.bucurica@umfcd.ro; 9 Department of Gastroenterology, University Emergency Central Military Hospital “Dr. Carol Davila”, 010825 Bucharest, Romania

**Keywords:** gut-brain axis, microbiota-gut-brain axis, enteric nervous system, interoception, gut feeling, short-chain fatty acids (SCFAs), vagus nerve, microbiome and mental health, microbiota, neurogastroenterology, microbiota and neuroinflammation, microRNAs, gut-brain communication

## Abstract

The gut-brain axis (GBA) represents an operant acting in a two-direction communication system between the gastrointestinal tract and the central nervous system, mediated by the enteric nervous system (ENS), vagus nerve, immune pathways, and endocrine signaling. In recent years, evidence has highlighted the pivotal role of the gut microbiota in modulating this axis, forming the microbiota-gut-brain axis (MGBA). Our review synthesizes current knowledge on the anatomical and functional substrates of gut-brain communication, focusing on interoceptive signaling, the roles of intrinsic primary afferent neurons (IPANs) and enteroendocrine cells (EECs) and the influence of microbial metabolites, including short-chain fatty acids (SCFAs), bile acids, and indoles. These agents modulate neurotransmission, epithelial barrier function, and neuroimmune interactions. The vagus nerve serves as a primary pathway for afferent sensory signaling from the gut influenced indirectly by the ENS and microbiota. Dysbiosis has been associated with altered gut-brain signaling and implicated in the pathophysiology of disorders ranging from irritable bowel syndrome to mood disorders and neurodegeneration. Microbial modulation of host gene expression via epigenetic mechanisms, including microRNAs, adds another layer of complexity. The gut has a crucial role as an active sensory and signaling organ capable of influencing higher-order brain functions. Understanding the MGBA has significant implications for new therapeutic interventions targeting the microbiome to manage neurogastroenterological and even neuropsychiatric conditions.

## 1. Introduction

The gut, a major interface with the external environment, is highly connected to the brain. Vivid communication between the gut and the brain plays key roles in regulating digestive and metabolic processes and is also involved in behavioral decisions [1,2]. Behavior results from the conjugated analysis of exteroceptive, interoceptive, and proprioceptive information [3]. Interoceptive and proprioceptive information are strongly emotionally codified and, consequently, highly personalized, self-referential, and impossible to interpret in a universal way [4].

The digestive functional responses to specific situations and the anticipatory sensations known as gut feelings—expressions of insight or intuition—mediate reciprocal signaling between the gut and brain [2,5]. Anxiogenic or stressful contexts may produce “rejecting” digestive symptoms, such as pain, nausea, and diarrhea [6,7]. In situations marked by uncertainty, especially within socially complex contexts, retrieving unconscious information can be instrumental in achieving a more accurate characterization of the specific circumstance [7]. Some visceral, vague sensations, resulting from the integration of visceral information at the subcortical level, are involved in preparing the most suitable behavior for a factual situation [8].

The capacity for accurate perception of internal physiological fluctuations, known as interoceptive sensitivity (IS), is considered an innate, constitutional trait that varies between individuals [9,10]. IS is directly correlated with emotion regulation ability [11]. If the inner bodily signals are properly felt, then they reinforce the stability of the material self [12].

A direct relationship exists between the intensity of bodily sensation perception and the intensity of emotional experiences [13]. People with high IS, estimated, for example, through their capacity to detect their heartbeats, describe intense emotional experiences [14]. The interoceptive function involves retrieving internal information, either generating quick behavioral responses to sensations or translating stressful events into digestive symptoms and functional changes, expressed as secretory, intestinal motility responses, or other somatic signs, such as palpitations [15,16]. Interoception may be viewed as a tool through which the brain maintains surveillance over bodily functions on one hand and anticipates future events on the other hand [16,17].

Establishing a reference for interoceptive capacity is challenging not only because the relationship between visceral sensitivity and specific organs’ activity remains unclear, but also because there is a scarcity of data quantifying interoceptive sensitivity (IS) across various target systems [17]. Most attempts are based on autonomous cardiac response, as measuring non-cardiac interoception is more challenging [15,17]. This is a concern related to cross-interoception performance, specifically the correlation between cardiovascular and digestive interoception [18,19]. Several studies on cross-cardiovascular, respiratory, and gastrointestinal IS concluded that IS is channel-specific rather than a general ability [20,21,22]. Overall, the cross-interoception performance remains a topic of debate; objective measures of interoceptive sensitivity do not consistently show correlations [21].

The relationship between the emotional state and gut function is a reality that is equally interesting to both gastroenterologists and psychologists and, up until now, probably understudied [23]. All intestinal components, including the microbiota, may influence higher cerebral function and behavior [24].

This happens, or at least is possible, because the microbiota, an ecosystem functionally integrated into the host, generates interoceptive signals [18,19,25]. Most signals originating in the digestive tract are not perceived because they are subliminal; only a small portion of intestinal signals is consciously perceived, most of which are those that influence a motor decision (such as feeding or defecation) [15]. The eponymous von Economo neurons, a special neuronal population found in the fronto-insular (FI) cortex and the anterior limbic area, play a special role in gut-feeling meta-representations [26]. These neurons express specific receptors, such as the serotonin 5HT2B receptor subtype and bombesin receptors, which are rarely expressed in other brain areas but are abundant at the intestinal level [26] (Allman, 2010). Von Economo neurons, large-sized neurons with a simple dendritic structure and faster-conducting axons, are highly engaged in intuition and rapid decisions in uncertain situations [26]. They possess anatomical traits particularly important for social decisions, when behavior must adapt quickly to real-time conditions, requiring a rapid response based on rapid intuition [26].

This review highlights the reciprocal relationship between the gut, the central nervous system, and the autonomic nervous system for the best behavior.

## 2. Materials and Methods

The aim of our review is to emphasize the significant association in the gut microbiota between brain function and gut microbiota composition, highlighting the interactive communication of the gut-brain axis. Due to the broad scope of information available in the literature, we developed a comprehensive search strategy to ensure the review encompassed the most relevant insights related to our topic. We searched two databases, Medline (via Pubmed Central) and Scopus, from inception up to 2024. The review only considered articles published in the English language. Although no formal protocol was registered or developed for this review, we included older publications when deemed relevant to the topic. The following search key was used: (“brain gut axis”[MeSH Terms] OR (“microbiota”[MeSH Terms] AND (“probiotics”[MeSH Terms]) OR (“enteric system”[MeSH Terms]) OR (“mental disorders “[MeSH Terms]). Duplicates were both automatically and manually excluded. An independent researcher (I.S.-F.) conducted the selection, and two others (A.-S.-N. and S.B.) solved disagreements. Two other independent evaluators (C.C. and S.-N.B.) extracted data. Studies involving both animal and human subjects were included. Articles written in languages other than English or those for which the full text was unavailable were excluded from the review.

## 3. Gut-Brain Crosstalk: The Gut-Brain Axis and Microbiota-Gut-Brain Axis Concepts

The key feature of gut-brain communication involves a wide signal exchange between the digestive tract and the central nervous system (CNS) in order to regulate specific responses mutually. Clinicians are familiar with intestinal transit disorders within irritable bowel syndrome (IBS) as an expression of top-down interferences and the bad mood within acute digestive illness as an expression of bottom-up communication. The information exchange on the gut-brain axis (GBA) is involved not only in short-term information about the actual food [27] but also in long-term feeding control [18]. Gut-innervating sensory neurons can detect food signals and non-food cues which are potentially harmful [28,29]. They are also involved in food selection behavior (preference or avoidance) [18].

The communication languages between the digestive tract and the CNS are represented by various neurotransmitters (NTs), hormones, immune triggers, or other molecules, such as microbiota-related signaling molecules [30,31]. The pathways are either the nervous fibers derived from the vagus nerve, spinal afferents, and autonomic nervous system (ANS) or the circulatory stream controlled by the blood–brain barrier (BBB) and the circumventricular organelles [30,31]. This bidirectional information exchange on the GBA is involved not only in digestive homeostasis but also in proper inclusion of the external world [18].

The contribution of microbiota in the communication between the gut and CNS and the central influence on gut microbiota is of high interest in current research. A concept (derived from the microbiota contribution) was imposed: the microbiota-gut-brain axis as a subsystem of the GBA [32,33]. The microbiota-gut-brain axis (MGBA) is a multimodal regulatory system that integrates the most extensive endocrine system with microbiota, a cell population that surpasses that of the human body. It also links two related systems on an ontogenetic basis, both of which are derived from the ectoderm, the CNS, and the enteric nervous system (ENS), while functioning through shared signaling pathways [34].

The microbiota is a cell population with highly plastic behavior that is significantly influenced by the external environment, with a common example being diet [35].

The approach to the MBGA concept is important not only to complete the understanding of the physiopathology of gut-brain crosstalk but also to realize that intervention to promote a “healthy” intestinal microbiota can lead to considerable modulation of this axis.

Many studies underscore the microbiota’s involvement in cognitive performance and disposition in various feeding behaviors [36], in physiological activities such as sleep quality [37], and also in cardiovascular and metabolic disease pathology [38].

Animal models help in understanding feeding interventions. For example, mice exposed to a high-fat diet (HFD) had both quantitative and qualitative changes in gut microbiota (the most important modification represented by a decrease in the *Lactobacillus reuteri* population), secretory changes (impaired oxytocin production), and social behavioral changes, with effects observed in both mothers and offspring. On the other hand, mice fed with an HFD that received *Lactobacillus reuteri* showed improved oxytocin production and sociability [39].

## 4. The Nervous Pathways in Gut-Brain Bidirectional Communication: Bottom-Up and Top-Down Signaling

The visceral information is transmitted to various CNS structures [40] in two ways: the spinal and the cranial pathways. On the homeostatic spinal pathway, nociceptive and thermic information and information from osmoreceptors and metaboreceptors reach the posterior and middle insular regions (the primary interoceptive cortex) and then proceed toward the anterior insular region, where afferent physiological signals are integrated with higher-order information and ultimately transmitted to the prefrontal cortex level [4,8]. The primary brain region responsible for interoception is the insular cortex [41].

**Figure 1:**
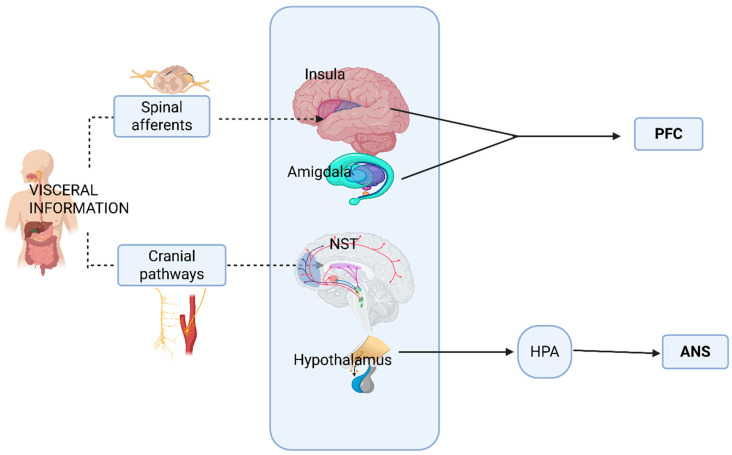
The central autonomic network structures, the autonomic nervous system, and the neocortex are highly connected and involved in visceral information processing. Visceral information is transmitted via the spinal and cranial pathways to various structures belonging to the central autonomic networks (CANs), between which reciprocal connections exist as well as connections with the hypothalamic-pituitary-adrenal (HPA) axis, the autonomic nervous system (ANS), and the prefrontal cortex (PFC). Visceral information transmitted through the spinal pathway reaches primarily the insular cortex, while information transmitted via the cranial pathway is directed to the nucleus of the solitary tract (NST).

On the cranial homeostatic pathway, the vagus (X) and glossopharyngeal (IX) nerves transmit information to the nucleus of the solitary tract (NST), where visceral inputs converge [42]. From the NST, visceral information is relayed to other components of the central autonomic network (CAN), which plays significant roles in feeding and social behavior (Figure 1) [4,40].

The CAN structures include many structures, such the periaqueductal gray matter (PAG), the parabrachial nucleus (PBN), the ventrolateral medulla, the nucleus of the solitary tract (NST), the raphe nuclei (including locus coeruleus), the hypothalamus, the insular cortex, the amygdala, and the primary and secondary gustatory cortex [42,43]. At the gustative cortex, there are identified receptors for all tastes (sweet, salted, sour, bitter, and umami), for inosine (present in meat and tuna), and for capsaicin [44,45,46].

Afferent vagus nerve fiber stimulation changes the levels of certain brain neurotransmitters (NTs), such as serotonin, glutamine, and GABA, as well as ANS activity, leading to behavioral and cognitive changes [47].

Within the limbic system, the hypothalamus occupies a central place. It interacts with the rest of the body through the two branches of the ANS: the sympathetic division (SNS) and the parasympathetic one (PNS) [48]. The hypothalamus intervenes in defensive reactions and stress-induced responses by stimulating the hypothalamus-pituitary-adrenal (HPA) axis and in motivated and reward-seeking behavior [49,50].

The hypothalamic arcuate nucleus is primarily involved in feeding control; the stimulation of some neuronal populations (such as neurons positive for the neuropeptide Y (NPY) and for Agouti-related protein (AGRP)) has orexigenic effects. Meanwhile, stimulation of another population of neurons existing within the arcuate nucleus, represented by neurons that express POMC, inhibits feeding [51].

The amygdala intervenes in the emotional coding of external stimuli and the regulation of negative effects [52]. The amygdala favors associations between inputs from different sensory areas and those originating from structures related to memory systems that carry past information and experience [53,54]. There are strong and bilateral connections between the amygdala and the prefrontal cortex (PFC) [53,54]. The amygdala receives strong sensory information, mainly from the insula and the association areas, and sends, in turn, meager projections to sensory areas [53,54].

The amygdala and the declarative memory system are strongly and reciprocally connected, similar to the way the amygdala connects with the hypothalamus and the brainstem, both of which are highly linked to the vagal system (Figure 1) [54].

## 5. The Leading Players in the Microbiota-Gut-Brain Axis

The capital networks involved in the MBGA include the CNS, the ENS, the ANS, and the HPA [55]. They key point of this axis is not represented by the components but the concept of interconnected systems with various effectors, including neuro-mediators, hormones, metabolic products, and immune effectors [56].

### 5.1. The Enteric Nervous System: “The Second Brain”

Intestinal innervation is represented by the sensory nerve fibers that originate from the nodose vagal ganglia and the dorsal root ganglia in the spinal cord. The ENS has an extremely complex structure [57,58] represented by a heterogeneous mixture of glial cells, the most numerous cells, and about 200 million neurons [59,60], which are two closely related cell populations [61,62,63].

Other cells with important roles in gastrointestinal (GI) tract physiology include the interstitial cells, situated near the smooth muscle cells [64].

Similar to the CNS, the ENS is described as a neural network capable of autonomous activity [65]. The ENS is structured with two main plexuses represented by the submucous plexus (Meissner) and the myenteric plexus (Auerbach). Both plexuses are connected to both ANS divisions, the SNS and PNS [66].

Both plexuses consist of three neuron types: the enteric intrinsic primary afferent neurons (IPANs), the motor neurons or efferent neurons, and the association neurons. The intrinsic sensory neurons (IPANs) regulate intestinal motility independently. They project to the mucosa, being activated by the epithelial sensors, excitatory and inhibitory motor neurons, and interneurons [60,66]. IPANs represent the most vulnerable ENS neuronal population, given the fact that they extend to the lamina propria of the intestine [60,67].

The enteric peristalsis is controlled by the motor neurons arising from the myenteric plexus, while absorption and secretion are regulated by the neurons emerging from the submucosal plexus [66].

The main classes of enteric neurons are represented by neurons that receive fast synaptic input, known as “S” neurons, and neurons with prolonged post-hyperpolarization, referred to as “AH” neurons [68]. IPANs belong to “AH” neurons, whereas motor and interneurons are “S” neurons, which are relatively depolarized at rest [69].

The ENS neurons express receptors for inhibitory and excitatory NTs such that most myenteric neurons express NO or are cholinergic [70,71], and the submucosal neurons express VIP or are cholinergic and rarely expressing nitric oxide synthase (NOS) [60]. VIP is the most important inhibitory NT of the ENS, and Ach is the most important excitatory one [60]. One key aspect worth mentioning is the difference between the ascending and descending pathways, as the former are mostly cholinergic while the latter pathways are primarily noncholinergic [72,73,74].

The IPANs belonging to the myenteric plexus extend to the lamina propria [60]. The IPANs, activated in response to luminal chemical stimuli and mechanical stimulation, are interconnected such that once the activity starts, the activity in the whole network to which they belong is self-reinforced [67]. The IPANs synapse with interneurons that either project to the submucosal plexus or have projections, ascending and descending, in the myenteric plexus, thereby engaging in autonomic response [60].

Aside from the IPANs, the interneurons synapse with the motor neurons and the enteroendocrine cells (EECs), providing other pathways for information to flow toward the ENS and the CNS, as well as the vasomotor and secretomotor neurons and the viscerofugal neurons [60].

The interneurons also conduct information toward the CNS, noting that the types of descending neurons are more prevalent than the ascending types [60,66,75]. Nevertheless, all ENS neurons are localized in the neighborhood of and make contact with the vagus and spinal afferents, providing bidirectional communication with the CNS via the ANS [76]. These connections may suggest that digestive tract activity, whether secretory or motor, correlates with higher brain functions to facilitate a coherent and systemic response.

Tuft cells are described as a chemosensory cell population resident [77,78,79]. They express various taste receptors and also release ACh [77]. They are located in the vicinity of the enteric nerves, especially those that express calcitonin gene-related peptide (CGRP) [80].

A consistent and continuous influence on the phenotypes and the integrity of enteric neurons is exercised by the gut microbiota such that modifications in a normal microbiota are followed by a decrease in the excitability of ENS neurons [26]. Pathogen agents alert the enteric neurons through different pathways mediated by toll-like receptors (TLRs): TLR1, TLR2, TLR3, TLR4, TLR7, and TLR13 [81,82,83].

One characteristic of the ENS is its inherent plasticity, which includes the capacity to learn and remember [60]. This trait is crucial for adaptation, as it generates new behaviors. The ENS demonstrated phenotypic plasticity not only in normal conditions, such as aging, but also in response to dietary changes, in disease states, and after probiotic treatment [84,85,86,87,88]. Some of these changes refer to alterations in receptor sensitivity, as well as neuron loss after exposure to an HFD [84,86]. In animal models, it has been demonstrated that synaptic inputs to “S” neurons are altered in intestinal inflammation, and the “AH” neurons are hyperexcitable [60,89].

Another trait of ENS is represented by neurogenesis, which is observed after treatment with a 5-HT4 receptor agonist or following microbial recolonization [90,91,92].

Changes in the microbiota are followed by significant alterations in ENS activity, involving both neurons and glial cells [28,92,93,94,95].

**Figure 2:**
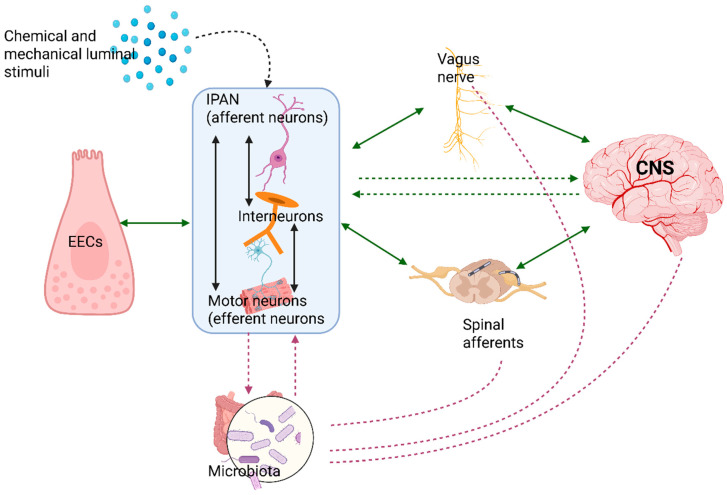
Gut–brain bidirectional interaction. There is a reciprocal relationship between the different neuronal populations of the enteric nervous system (intrinsic primary afferent neurons (IPANs), interneurons, and motor neurons). There is also a direct relationship between the enteric nervous system and the vagal and spinal afferents, providing bidirectional communication with the central nervous system (CNS) via the autonomic nervous system (ANS). The gut microbiota has a dynamic and continuous influence on the integrity and function of the enteric nervous system. Of particular note is the bidirectional relationship between interneurons and enteroendocrine cells (EECs) as well as the CNS structures, noting that descending neurons are more prevalent than ascending neurons.

“AH” neurons’ activity is influenced by the microbiota and microbial metabolites, among other factors [60]. Some authors observed that “AH” neurons belonging to the jejunal myenteric plexus become relatively unexcitable in germ-free mice [93]. The probiotic species *Lactobacillus reuteri*, *Lactobacillus rhamnosus* (JB-1), and *Bacteroides fragilis* lead to an increase in “AH” excitability [94,95,96]. Meanwhile, the *Bacteroides longum* species has the opposite effect [88] (Figure 2).

### 5.2. The Enteroendocrine Cells

The EECs, a minority cell population representing only 1% of all epithelial intestinal cells, have the most important secretory role [60,97]. They are activated by various intestinal stimuli, including nutrients and non-nutrients such as bacteria and their metabolic products, end products (for example, indole resulting from tryptophan metabolism), and substances that are detrimental to health [98,99]. Once activated by the luminal stimuli, EECs secrete hormones and neurotransmitters (NTs) that act locally in a paracrine mode, influencing the activity of adjacent cells and ENS components, and also at the central level through the secretion products delivered into the bloodstream [98,100,101]. Local communication between the EECs and the enteric afferent neurons is possible because the EECs are connected not only physically (through neuropodes, pseudopod-like basal cytoplasmic processes) but also functionally (through the secreted peptides) with the enteric neurons [100]. Three-dimensional (3D) electron microscopy and confocal imaging revealed a close “synaptic” relationship between the EECs and nerves, as well as between the EECs and glia [98,102,103]. As long as enteric nerves and glia do not penetrate the gastrointestinal epithelium, they rely on signals provided from the activated EECs in response to mechanical and chemical stimuli, such as release peptides, serotonin, and melatonin [98,104]. Moreover, the prion protein and α-synuclein, which are present in the EECs, may represent a transmission pathway from the gut to the brain via either the ENS or the vagus nerve [105,106].

EECs are short-lived, undergoing continuous renewal throughout life [18]. It is not clear whether the EECs maintain or change their contacts during EEC movement and maturation [18]. The EECs represent the nodal point between the molecules in the GI lumen, the IPANs, and the afferent extrinsic neurons.

EECs differ structurally and functionally, depending on the digestive tract segment.

From a morphological point of view, the EECs are of two types: the open type (equipped with microvilli, which detect the luminal content) and the closed-type (localized in basal membrane proximity without contact with the enteric lumen, activated indirectly by the endoluminal contents) [107].

The enterochromaffin cells (ECs), which are L-type enteroendocrine cells, are the most extensively studied cell population within the entero-endocrine system. The ECs, representing the body’s most numerous endocrine cells, are abundant in the distal part of the ileum and within the large intestine, where there is a higher density of bacterial taxa [60,108]. This is not a coincidence but rather a coexistence with functional significance. The reverse also sustains this thesis; serotonin levels are significantly reduced in germ-free mice [109,110]. In the same sense, tryptophan transcription is enhanced after colonization of GF mice or after exposure of the human EC cell line to short-chain fatty acids (SCFAs), which are bacterial metabolites of dietary fiber fermentation [110,111].

ECs’ secretion responds to mechanical stimulation or chemicals, such as glucose and fatty acids [108]. ECs release serotonin in response to stimuli other than nutrients, such as TRPA1-activating irritants, catecholamines, and SCFAs [98].

L-type cells are activated in response to various stimuli according to the digestive tract to which they belong. In the proximal gut, the EECs are stimulated by various molecules, including carbohydrates (CHs), long-chain fatty acids (LCFAs), and monoacylglycerols. In the distal gut, EECs are activated almost exclusively by bacteria-derived metabolites, including short-chain fatty acids (SCFAs), lipopolysaccharides (LPS), secondary bile acids, and indole [101]. L-type cells produce glucagon-like peptide 1 (GLP-1) and peptide YY (PYY), two neuropeptides with key roles in regulating intestinal transit and appetite [112]. In the L cells located in the distal part of the gut, GLP-1 and PYY are simultaneously expressed with INSL-5 [113]. The density of L cells increases as they progress to the more distal regions of the intestine [114]. In the proximal small intestine, a more significant proportion of L cells co-express cholecystokinin (CCK), unlike the L cells belonging to the distal part of the bowel, where they co-express PYY [115].

EECs synthesize various molecules, including gut hormone peptides YY, CCK, GLP1, ghrelin, gastric inhibitory polypeptide (GIP), and serotonin [97]. Some of them, such as CCK, GLP-1, and PYY, are released after and suppress feeding. Others, such as GLP-1 and GIP, stimulate insulin release [116]. Five principal EEC lineages have been identified, leading to the synthesis of GIP, ghrelin, serotonin, somatostatin, or a mixture represented by GLP-1, CCK, or neurotensin [117]. The nutrients trigger both common (satiety) and nutrient-specific responses [18]. The EECs frequently co-express more than one hormone, such as CCK, PYY, and GLP1, so that each nutrient class triggers the release of a mixture of gut hormones [97].

In some brain areas, receptors for some gut hormones were recognized, suggesting a place for gut hormones in behavior [18]. For instance, GLP-1, acting at hypothalamic and caudal brainstem levels, induces satiety and nausea-related behaviors, which are brainstem responses [118,119].

Another example is represented by the receptors for GLP-1 and PYY, which are identified on the vagal afferents and enteric neurons and within the CNS [120,121].

ECs integrate the dietary, immune, mechanical, and microbial signals, releasing serotonin and initiating motor reflexes [60].

Serotonin synthesis is regulated within the large bowel by the commensal spore-forming bacteria [109].

ECs sense bacteria through an ion channel (the cation channel Piezo 1), which acts as a sensor for single-stranded RNA, triggering serotonin production by the ECs present in the small and large bowel [122]. The Piezo 2 ion channel is expressed by various types of EECs but mostly by ECs [123,124]. ECs highly express the transient receptor potential ankyrin A1 (TRPA1) channels for irritant substances and also express olfactory receptor Olfr558 (418) for microbial metabolites. In zebrafish, the bacteria *Edwardsiella tarda* activate ECs through the TRPA1 receptor [125]. Additionally, the microbial catabolites of dietary tryptophan are detected by the ECs, resulting in the release of serotonin and the activation of cholinergic nerves [60].

ECs can be activated by catecholamines because they possess α2 receptors [98].

ECs secrete over 95% of the whole-body serotonin [126,127]. Vagal afferents highly express the serotonin receptor HTR3A [128].

The principal amount of the secreted serotonin is delivered at the intestinal submucosa level, with only small amounts reaching the intestinal lumen [129]. Under the action of certain bacterial strains, Clostridia taxa for instance, or in response to specific microbial metabolites, serotonin synthesis increases [111,130].

Through serotonin synthesis, the ECs modulate vagal afferents and the inflammatory intestinal response [131].

EECs express, on their luminal side, many sensory receptors like type T1R taste receptors (sensing sweet and umami tastes) and type T2R (sensing bitterness) [132,133]. Postprandially, especially after high-fat and high-carbohydrate meals, CCK induces satiety through the activation of T1Rs [134]. Additionally, EEC activation has an important place in defensive responses triggered by exposure to hazardous substances, as it facilitates binding to T2Rs and promotes the secretion of ghrelin [135,136].

An interesting fact is that T2Rs are also used by the microbiota [101]. T2R38, the receptor responsible for bitter tastes, is expressed by the EECs that secrete anorexigenic hormones, such as GLP-1, CCK, and PYY, acting to decrease both feeding and food motivation and also contributing to conditioned taste aversion [101]. Bitter taste receptors from the EECs and microbiota participate, through learning, in a conditional aversive response [101]. In overweight and obese people, T2Rs are overexpressed, and T1Rs are decreased [101,133]. The feeding stimuli, in addition to their quality as first-order stimuli, acquire the quality of second-order stimuli through the learning of connections between various flavors and post-ingestion effects [137,138]. The fact that post-ingestive signals may override innate signals is the cornerstone for food and nutrition education; it can be learned to prefer bitter but healthy nutrients and to avoid sweet but unhealthy foods [139,140].

EECs regulate food intake not only by activating taste receptors but also by synthesizing CCK [97]. Low doses of CCK contribute to satiety and food rewards and are directly linked to food preference [141]. On the contrary, discharging high amounts of CCK may result in aversion [141]. Interestingly, odorant receptors, expressed by many EEC types, are present in the digestive tract and activated in response to the odorants produced by microbiota [108]. Odorant receptor activation results in serotonin secretion and release by ECs [108].

Overall, taste and smell receptors at the digestive tract level support the idea that the gut is a vast sensory organ. Its behavior reminds one of the central behavior in response to first-order or second-order stimuli: approach or reject [142].

Apart from the previously mentioned receptors, some EEC types express free fatty acid receptors (FFARs) for SCFAs and receptors for microbial products [143,144]. Activation of FFAR1-3 receptors stimulates CCK release (resulting in satiety) and GLP-1 and PYY release [145].

EECs express TLRs that detect microbiota signals and recognize some bacterial products, such as LPS [30]. One key fact is that the information provided by the TLRs is further conveyed to both intrinsic and extrinsic primary afferent neurons [30].

**Figure 3:**
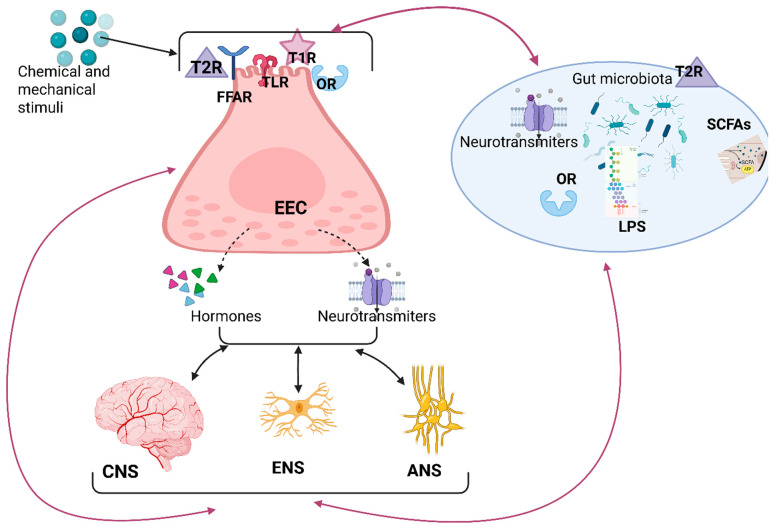
Bidirectional signaling between microbiota, enteroendocrine intestinal system, and nervous system. Enteroendocrine cells (EECs) release various hormones and neurotransmitters (NTs) that exert local effects on different components of the enteric nervous system (ENS), the autonomic nervous system (ANS), the microbiota as well as other EECs. They also have distant effects via the bloodstream, including on the central nervous system (CNS). EECs express various receptors such as taste receptors T1R (which sense sweet and umami flavors), T2R receptors (which sense bitter tastes), and odorant receptors (ORs) for odorants synthesized by the microbiota, as well as receptors for other microbiota-derived substances (such as free fatty acid receptors (FFARs) for short-chain fatty acids (SCFAs) and Toll-like receptors (TLRs) for lipopolysaccharides (LPSs)). For certain hormones and neurotransmitters synthesized by EECs, receptors are found on structures belonging to the ENS and ANS and also at the central level. The microbiota also releases neurotransmitters and various active metabolites, some of which do not cross the blood–brain barrier (BBB), such as serotonin, dopamine, and GABA, and others that do cross the BBB, exerting central effects, such as tyrosine (Tyr), tryptophan (Trp), and phenylalanine.

A brief concluding remark is that sensory information is processed and regulated not only at the level of the oral cavity but also at the levels of the gut and microbiota [146]. All visceral and gustatory information flows to the insular cortex, the anatomical location where interoceptive information is integrated (Figure 3) [146].

### 5.3. The Autonomic Nervous System

The enteric neurons receive inputs from both divisions of the ANS, such as from the other enteric neurons [60].

Spinal and vagal afferents play a role in maintaining the homeostatic regulation of gut function [60].

The cell bodies of vagal afferents are localized in the nodose ganglia, and the terminals end in the intestinal mucosa and smooth muscle. A special role is played by the afferent vagal terminals in the myenteric plexus, known as intraganglionic laminar endings (IGLEs), which are mechanosensitive and also responsive to enteroendocrine peptides, such as CCK [60,147]. IGLEs integrate the chemical signals with the mechanosensory neural signals [60]. A unique characteristic of the nodose ganglion neurons is their exceptionally high plasticity, which enables them to modify the expression of certain neurotransmitters and receptors in response to food and obesity [148]. The vagal afferents carry information that is incredibly important for interoceptive sensations, including mood and food reward value [149,150,151].

The spinal primary afferents are strongly involved in gut microbiota composition [152].

The vagus nerve contains a majority of afferent fibers and also carries efferent motor fibers. The vagus nerve is often referred to as “the sixth sense” due to its role in interoceptive awareness [153]. The cell bodies of spinal afferents are localized in the dorsal root ganglia, and the terminals end in the submucosa, mucosa, myenteric plexus, and the circular muscle levels [154]. Vagal afferent terminals, which are chemo- and mechanosensitive, innervate all gut layers until the subepithelial level without exceeding the gut epithelium so that the vagus terminals do not have direct contact with food or the microbiota [47]. This suggests that the information that flows through the vagus nerve is multifaceted and braided, being initiated by chemical and mechanical analysis in structures other than the vagal endings [18]. Neurons belonging to the ENS, vagal afferents, and neurons from the CNS express receptors for substances synthesized by the EECs [120,121]. Vagal efferents convey visceral information to the central level. Stimulation of the gastrointestinal tract’s mechanosensory vagal and dorsal root ganglion (DRG) neurons during feeding or drinking induces the sensation of fullness [18,155]. Bariatric surgery may adjust the sensory thresholds of gut mechanosensory neurons [16]. Some sensory vagal neurons project to the area postrema, being involved in vomiting reactions or aversive behavior [18]. Studies in mice have shown that stimulation of area postrema neurons induces flavor avoidance, whereas ablation of this neuronal population removes behavioral reactions to various nausea-inducing toxins [119,156]. GLP1r agonists acting on the area’s postrema neurons leads to behavioral aversion [119].

The significant contribution of the vagus nerve to visceral and central homeostasis must be underscored. The vagal pathway is viewed as a two-way communication route linking the periphery with the central system that drives the best responses [18]. The gut microbiota produce signals sent to the brain through vagal afferents [157]. The ingested food is characterized by taste, odor, temperature, and texture. After it is swallowed, the information is transmitted to extrinsic nerves, mostly to the vagus nerve, along the length of the digestive tract [18]. Extrinsic motor neurons, as well as some extrinsic sensory neurons, are mostly connected with the enteric neurons [18].

The vagus nerve presents different sensory neuron types, including mechanoreceptors (IGLEs and intramuscular arrays) and chemoreceptors, such as mucosal receptors in the intestinal villi and crypt endings [18]. Recent data demonstrated significant diversity among transcriptome-defined vagal and spinal sensory neurons in the digestive system [37,158].

As long as vagal afferents do not surpass the epithelium, the extrinsic chemosensory neurons act as second-order neurons. These neurons receive sensory inputs primarily from EECs and enteric neurons, along with signals from other cells, like tuft cells or immune cells [18].

The vagus nerve conveys the information to the CNS. However, the speed of this transmission varies, depending on the source of the vagal stimulus. Thus, vagal terminal exposure to certain microbial metabolites, such as SCFAs, generates rapid responses, typically within seconds. Meanwhile, the intraluminal administration of probiotics without direct contact with vagal afferents results in delayed (up to a minute) vagal terminal activation [159].

The vagal mechanosensory neurons are primarily slow-conducting C fibers that are capsaicin-sensitive [128]. They are in close contact with the enteric neurons. Gut mechanoreceptors frequently co-express receptors for several gut satiety hormones, such as CCK, GLP1, and PYY [128]. Vagal sensory neurons responsible for detecting gut distension usually differ from mucosal chemosensory neurons, and they typically do not activate in response to nutrients [128]. Additional studies are needed for details. The vagal mechanosensory neurons trigger rapid and persistent responses, even in the absence of extracellular calcium [160]. Stimulation of vagal GLP1r neurons leads to reduced feeding, whereas activating vagal OXTR neurons has a more dramatic effect on feeding reduction [158,161]. A decrease in water intake is obtained only through activation of OXTR neurons [158].

Vagal and spinal afferents send collaterals that innervate the ENS, and through these collateral branches, the information provided by ENS neurons is transmitted toward along the CNS [18,162]. Vagotomy is followed not only by significant secretory and functional intestinal consequences but also by central responses. Subdiaphragmatic vagotomy results in a decrease in body weight, which affects absorption, digestion, and feeding behavior [163,164]. Vagotomy, proposed some time ago for peptic ulcer cure, was followed by an increase in psychiatric-related disorders [165]. Experimental studies in taste-blind mice showed that subdiaphragmatic vagotomy abolishes post-ingestive sugar preference. As a result, the vagotomized mice showed a preference for sugar instead of artificial sweeteners by seeking an alternative reinforcement pathway for sugar [166,167]. The strong connection between the epithelial cells and vagal terminals is suggested not only by vagal terminal localization in the proximity of the epithelial cells but also by the direct connections between the EECs and the vagal terminals and through the existence of the receptors for peptides and hormones released by the EECs on lamina propria vagal terminals [18]. Some vagal sensory neurons express receptors for hormones synthesized by the EECs [128]. Some authors are not categorically relative to the communication between the EECs and the vagal afferents [18].

An intriguing characteristic of vagal terminals is their significant plasticity, as indicated by the variation in the density of vagal receptors for orexigenic and anorexigenic peptides based on the host’s fasting-satiety status [18,168].

The intestinal epithelium’s exposure to various nutrients (carbohydrates, fats, and amino acids) is followed by EEC stimulation and the release of hormones and neurotransmitters, which signal both the ENS and vagal afferents [18]. The basis of gut sensory chemoreception is represented by the “conversation” between the EECs and the vagal afferents, a complex process modulated by the microbiota [169]. Vagal afferents are activated indirectly through the connections between the ENS and the vagus nerve [18].

**Figure 4:**
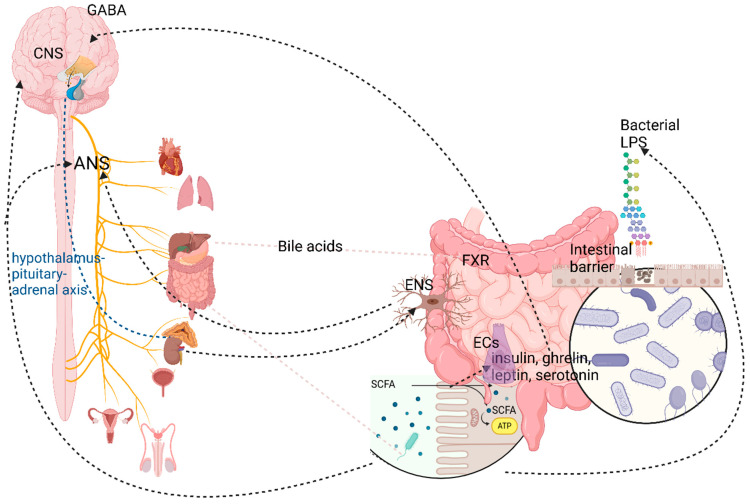
Gut-brain axis and metabolite interaction. CNS = central nervous system; ANS = autonomous nervous system; ENS = enteric nervous system; ECs = enterochromaffin cells; SCFA = short chain fatty acids; LPS = lipopolysaccharides; FXR = farnesoid receptors; GABA = gamma-aminobutyric acid.

The microbiota either release microbial components (like LPS), for which there are TLR receptors on the EECs, or release metabolites (like SCFAs), for which there are receptors on the EECs [47,170]. Moreover, the vagus nerve expresses TLR4 receptors for LPS and also receptors for short-chain fatty acids (SCFAs) (Figure 4) [47]. Despite the absence of direct contact between the gut microbiota and vagal afferents, the vagus nerve represents the primary sensory pathway for visceral information, including that from the microbiota, to reach the CNS [171,172]. This statement is sustained by the observation that after vagotomy, the beneficial effects of *Lactobacillus* spp. and *Bifidobacterium* spp. on cognitive functions are abolished [173]. Similarly, the positive effects of probiotic ingestion on cognition are only evident when the vagus nerve is intact [173].

The EECs are central to detecting luminal signals, including those from the microbiota. It is necessary to highlight the intricate interactions among the ENS, EECs, microbiota, and neurons; the gut microbiota regulates hormone production, which interacts with the EECs, generating signals that react with the enteric nervous system (ENS) [174,175,176].

Overall, EECs can be categorized as polymodal chemosensors, which integrate external and internal information to transmit to nerve terminals [32].

SNS stimulation, acting on cholinergic transmission and contracting the smooth muscle cells, decreases intestinal secretion and motility [67].

The top-down regulation consists of three levels of interaction: the central nervous system (CNS), the autonomic nervous system (ANS), and the enteric nervous system (ENS). The inverse route begins at the ENS, which converts signals from the GI tract into nerve impulses sent to the CNS. The ANS, through its branches, controls the GI tract, including functions such as motility, secretion, and blood flow. Furthermore, it is suggested that ANS has an impact on epithelial stem cell proliferation [177,178].

Certain structures in the central nervous system (CNS), such as the amygdala, the hypothalamus, and the nucleus of the solitary tract (NTS), play a role in this regulation by controlling the gastrointestinal (GI) tract through the sympathetic and parasympathetic nervous systems [34]. The HPA regulates how stress impacts the GI tract. Intestinal cells can undergo downregulation and upregulation, creating various signaling molecules, some of which can penetrate the blood–brain barrier to access the central nervous system (CNS) bloodstream [34]. Most neurotransmitters generated by the microbiota, such as serotonin, dopamine, and gamma-aminobutyric acid, are unable to cross the BBB. The microbiota-brain axis may shed light on a range of disorders affecting the nervous system, gastrointestinal tract, and liver. However, further investigation is warranted [179].

## 6. The Gut Microbiome (Microbiota)

The intestinal microbial flora is primarily represented by a rich bacterial population, which is essential for maintaining intestinal integrity and certain metabolic functions [180]. The microbiota consists of a diverse community of microorganisms belonging to a particular environment, including the human body or a certain part of the body [181]. In other words, the gut microbiota constitutes one of the most extensive interfaces between individuals and environmental factors [182]. In contrast, the genomes of microorganisms living in a particular environment represent the microbiome. However, this definition may not be as straightforward as it appears [181].

The Human Microbiome Project characterizes the human microbiome as the complete ensemble of microorganisms inhabiting the human body [183]. This diverse collection includes eukaryotes, archaea, bacteria, and viruses (NIH Human Microbiome Project—Home, n.d.) [184]. A panel of international experts revisited the definition of microbiota and microbiome and concluded that the term microbiome covers the microbiota (the living organisms) and their “theatre of activity”, including microorganism-related structural elements, nucleic acids, metabolites, signal molecules, and mobile genetic elements, such as viruses and phages. This “theater of activity” includes the surrounding environmental conditions [181]. Some authors consider the human microbiome to be the “last human organ” or “the hidden organ”, an organ with its physiology and pathology [180,185].

Most of the time, humans exist in symbiosis with the microbiome, which serves numerous functions: food digestion and nutrient assimilation, synthesis of various metabolites, host defense by preventing the colonization of pathogenic microorganisms, removal or metabolism of certain substances, including drugs and some toxins, regulation of the immune response including the training of cells, and the overall maintenance of gut homeostasis [186,187,188]. The microbial flora is a system continuously exposed to changes in the external environment and constantly communicates with the host organism [189,190,191]. Directly and through various products of microbial metabolism, the gut microbiota influences cognitive functions, mood, and behavior [192].

The human gut contains 10^13^–10^14^ microorganisms belonging to more than 40,000 species. The dominant phyla are Firmicutes (*Lactobacillus* spp. being the most numerous, but also including the *Clostridioides*, *Enterococcus*, and *Faecalibacterium* genera) and Bacteroidetes (which includes the *Bacteroides* and *Prevotella* genera) [193,194,195].

Other phyla, represented in lower concentrations, include Actinobacteria (such as *Bifidobacteria)*, Proteobacteria, Verrucomicrobia, and Euryarchaea [193].

The bacterial population vastly outnumbers the nucleated cells of the human body, encompassing approximately 150 times more genes than the human genome [196].

This data was mandatory for many studies centered on the beneficial effects of a healthy microbiota and, on the other side of the coin, the implications of dysbiosis in the pathogenesis of diverse diseases, including metabolic syndrome, non-communicable diseases, and various neuropsychiatric conditions [197]. A healthy microbiota appears to be unique to each individual and is linked to both maternal microbiota and diet [198,199]. Microbial diversity increases in the first years of life, usually from birth to the age of 3–5, until the adult-like microbiota is established [200,201].

The spectrum of intestinal flora typically varies with age; in elderly individuals, the levels of *Lactobacillus* and *Bifidobacterium* are lower, and the ratio between Firmicutes and *Bacteroides* is low [202,203].

The quantity and diversity of the intestinal flora also vary in response to other factors, including diet, psychological state (such as stress), medicinal interventions (like antibiotics), and digestive system infections [204,205].

Studies have shown that people who share a geographical area have similar gut microbiota compositions [206]. Differences appear to be related specifically to the concentration and number of different bacteria and less to the diversity of bacterial species [206]. Throughout the evolution of the human race, considerable changes have occurred in the gut microbiome [207]. The world population is currently undergoing a shift in microbiota composition toward the Western-associated *Bacteroides*, *Blautia*, and *Bifidobacterium* clusters [208,209]. For example, the HELIUS study showed that the microbiota of second-generation Moroccans and Turks that moved to Holland suffered an important shift from a cluster rich in *Prevotella copri* and *Prevotella stercorea* (members of the *Prevotella* cluster, which is associated with an increased fermentative capability and healthy BMI) toward the classical Western *Bacteroides*/*Blautia*/*Bifidobacterium* cluster, which is associated with diseases of affluence, namely the “big four”: cancer, diabetes, and cardiovascular and pulmonary diseases [208].

At the same time, such shifts were not observed in the population originating from South Asia, including the African Surinamese; in these populations, the *Bacteroides*/*Blautia*/*Bifidobacterium* cluster was dominant. Nevertheless, an increase in some species associated with obesity was noted [208].

Some authors have attempted to classify gut microbiota into three distinct genotypes of intestinal bacteria based on the predominant bacterial species: *Bacteroides* (found particularly in individuals with diets high in protein or fat), *Prevotella* (common in those with carbohydrate-rich diets), and *Ruminococcus* [32].

The gut microbiome participates in immune system development, contributing to the avoidance of hyperactivity against non-pathogenic germs and even food antigens [210]. Azad et al. demonstrated that a lack of diversity in gut microbiota characterized by an elevated Enterobacterales/Bacteroidaceae ratio, especially during infancy, contributes to food sensitization and, subsequently, to the development of food allergies [210]. Between persistent low levels of *Bacteroides* throughout childhood and peanut sensitization, a relationship was found [211].

Dysbiosis refers to alteration of the commensal flora, characterized by a low bacterial richness and the outgrowth of pathogenic species [212]. The administration of antibiotics that alter the intestinal flora is associated with long-term functional effects on the ENS, spinal cord, and brain [213,214]. The absence of microbiota experimentally demonstrated a decrease in IPAN excitability, as the microbiota also regulates the enhancement of the ENS and the regeneration of neurons belonging to the ENS [93,215]. Thus, the microbiota plays a major role in gut-brain communication as it is considered an alternative pathway to the vagal one, through which various microbial metabolites convey information to central nervous networks [216,217].

The absence of microbiota experimentally proved a decrease in IPAN excitability. The fact that some species belonging to the normal microbiota, such as *Lactobacillus* spp. and *Bifidobacterium* spp., may have central protective effects, even after vagotomy, represents another proof of the significant function in gut-brain communication [218]. The microbiota interacts with the GBA through several mechanisms, namely modulation of intestinal barrier permeability, action on the enteric nervous system (ENS) through neurotransmitters (NTs) and active released metabolites, expression of hypothalamic genes involved in synaptic plasticity, and generation of nitric oxide (NO) and hydrogen sulfide, which interact with capsaicin receptors present on nerve fibers [219,220,221].

The microbiota acts locally on enteric cells and vagus nerves to transmit composite signals to the brain quickly [222]. In addition, the microbiota intervenes in neuroinflammation, a process linked to various neurodegenerative diseases such as Alzheimer’s disease through bacterial products, including endotoxins and lipopolysaccharides (LPSs), which activate the peripheral immune system [223]. Moreover, the changes in the microbiota and their active metabolites synthesized by the bacterial population have behavioral consequences, and these effects are also observed in patients with various psychiatric disorders, like depression or autism spectrum disorders [224].

Several experimental and clinical observations suggest that gut flora influences the activity of the CNS [225]. Changes occurring in the amygdala and hippocampus might be explained, at least in part, by the involvement of gut microbiota in behavioral functions [225]. Alteration of brain functions has been proven in germ-free animals, along with improved brain and behavioral functions following the transfer of microbiota samples from healthy animals to germ-free animals [226,227]. Microbiota alterations determine HPA hyper-reactivity and the subsequent cognitive deficit [228,229]. The administration of prebiotics, probiotics, or psychobiotic foods improves cognitive functions [230]. Exposure to psychological stress, even for a fairly short duration (2 h), alters the diversity of the microbiota through the SNA pathway and the activation of the HPA but also through action on the effectors: the ENS, EECs, and intestinal immune cells [231]. Receptors for certain enteric neurotransmitters have been identified on the surface of bacteria, where their activation induces a range of functional changes in bacterial behavior [231,232]. Stress has effects on the intestinal permeability, as well as on the composition of intestinal mucus [218]. In addition, acute stress stimulates mast cell degranulation, which releases mediators, thus causing intestinal muscle dysfunction [233]. The expression of virulent bacteria such as *Pseudomonas aeruginosa* and *Campylobacter jejuni* is stimulated in response to stress [234,235].

The impact of various types of microbial supplements on anxiety and stress-related disorders has been extensively evaluated in recent studies [173]. Studies have shown a direct influence of the gut microbiota on major brain regions involved in emotional and behavioral responses. Administration of *Bifidobacterium longum* in IBS patients reduces emotional reactivity, with decreased amygdala and fronto-limbic activity [236]. High-dose administration of specific bacterial strains appears to not only modulate the clinical manifestations of anxiety-related disorders but also influence stress responses through the activation of distinct inflammatory pathways. Reduced levels of cortisol and pro-inflammatory cytokines have been reported following probiotic intervention, as shown by Önning G et al., Boehme M et al. (2023) [237,238].

Table 1 provides a summary of the most recent studies examining the effects of various gut-targeted interventions and their potential therapeutic impact on stress-related disorders.

Despite the fact that the microbiota does not pass the epithelium and has no direct contact with the nerve endings of the IPANs or the vagal afferent endings, as mentioned before, its effects are exerted through microbial-associated molecular patterns (MAMPs) and various microbial metabolites, some of which have neuroactive properties [243,244,245]. Moreover, bacteria communicate through hormonal, neurochemical, and metabolic pathways, as well as via immune system signaling and hormonal regulation [243]. An alternative mechanism of microbial communication is quorum sensing, a process through which bacteria modulate their gene expression in response to signals from neighboring microbial cells or the host organism [244]. This complex mechanism, through which bacteria manage to coordinate intricate processes at a molecular level, is driven by specific molecules known as quorum-sensing molecules (N-acyl homoserine lactones (AHLs), γ-butyrolactones, oligopeptides etc.), emphasizing the gut microbiota’s role as “the hidden organ” due to its dynamic interactions with these various cognitive behavioral or humoral signaling networks [245,246]. Cell-to-cell communication has a crucial role in controlling bacterial pathogenicity and the production of bioactive metabolites [247]. Moreover, recent studies have highlighted the intricate nature of bacterial communication and its role in the pathogenesis of various diseases, particularly in inflammatory bowel disease and certain types of cancers [248,249]. *Enterococcus faecalis* exacerbates local intestinal inflammation via activation of quorum-sensing proinflammatory pathways, worsening local enteritis in rodent subjects [249]. Additionally, Cai X et al. reported a high abundance of *Akkermansia muciniphila*, a commensal bacterium known for producing inosine and the Amuc protein, which enhances the efficacy of immune checkpoint inhibitors in colorectal cancer [250]. The contribution of the microbiota to the GBA is wide.

Firstly, the microbiota stimulates the production of several neurotransmitters (NTs), including GABA, Ser, melatonin, histamine, acetylcholine (Ach), norepinephrine (NA), and dopamine (DA), which play various roles in nerve signaling and intestinal physiology. For example, certain *Lactobacillus* species activate the synthesis of acetylcholine (ACH) and gamma-aminobutyric acid (GABA) [221,251,252]. The decrease in acetylcholine (Ach) secretion and cholinergic signaling is followed by adrenergic signaling, which is causally related to the inflammatory alterations in the GI tract [215]. Microbiota stimulates the synthesis of serotonin by upregulating the expression of tryptophan hydroxylase 1 [215]. Certain germs, such as *Lactobacillus rhamnosus*, can lead to both an increase in GABAB receptor levels (which cause slowly generated and prolonged inhibitory signals) and a decrease in GABAA receptors (which produce fast inhibitory signals) [173,253]. Many studies have linked dysbiosis to several neurological and psychiatric conditions, including depression, schizophrenia, autism spectrum disorder, bipolar disorder, and obsessive-compulsive disorder [224].

Furthermore, the microbiota synthesizes precursors of neurotransmitters (NTs), such as phenylalanine, tyrosine (Tyr), and tryptophan (Trp), which can cross the BBB [254]. Typically, the level of NT precursors in the brain is low, and thus the possibility of supplementing NTs at the central level seems important in certain situations [255]. For example, in acute stress situations, Tyr prevents the depletion of NA from neurons in the locus coeruleus, improving the stress response (attention and alertness) [256]. The ability of certain precursors of neurotransmitters synthesized by the microbiota to pass through the blood–brain barrier indicates, on one hand, the presence of a functional reserve and also emphasizes the microbiota’s role as a regulator of the central response.

Nonetheless, SCFAs, secondary bile acids, and other bioactive molecules (such as branched-chain amino acids, BCAAs, endocannabinoids, and peptide glycans), result from the digestion of various compounds present in food, such as fibers, under bacterial action [32].

SCFAs have a central role in gut-brain signaling and functional modulation [257]. They are represented in a 95% proportion by acetate, propionate, and butyrate, with different effects at the central and peripheral levels [258]. The Bacteroides phylum mainly produces acetates and propionates, while bacteria of the genus *Firmicutes* produce butyrate [203]. At the intestinal level, SCFAs assist in upholding the integrity of the enteric barrier [259]. Experiments have shown that in dysbiosis, where the synthesis level of SCFAs is low and the intestinal permeability is increased, various microbial metabolites, including corticosterone, pass into general circulation, activating the HPA axis [260]. Furthermore, SCFAs restore neuronal loss [92]. Lower levels of SCFAs associated with dysbiosis may have, as a consequence, a loss of enteric neurons [60,215]. Released in the circulatory stream, SCFAs reach the cerebral level, acting at the CNS level as signaling molecules, intervening in the modulation of neuronal, glial, and BBB functions [257,261]. SCFAs transferred at subepithelial levels determine the modulation of ENS activity [262]. In dysbiosis, both the decrease in production of SCFAs and the loss of neurons belonging to the ENS were noted (Table 2) [92].

SCFAs are involved in the production and release of serotonin and catecholamine as well as other substances by activating their receptors (free-fatty acid receptor 2 and 3 (FFA2 and FFA3, respectively)), which have a higher density in the distal region of the small bowel and in the colon [131,275,276]. Additionally, higher levels of SCFAs are correlated with higher choline levels in the ACC [236,258]. Notably, choline has been shown to confer protection against fat accumulation, enhance the abundance of anti-inflammatory gut microbiota, modulate central nervous system (CNS) processes both directly and indirectly, and stimulate cognitive and behavioral functions [259,277]. Furthermore, the SCFAs lead to an increase in the level of dopamine (DA) both by activating tyrosine hydroxylase and by decreasing the level of beta-hydroxylase, the enzyme responsible for the peripheral conversion of DA to norepinephrine (NA) [214,259]. The other neuroendocrine SCFA influence is exerted by activating the synthesis of satiety modulators such as insulin, ghrelin, leptin, and Ser from ECs [261,278]. SCFAs intervene in satiety by stimulating the release of GLP-1 and PYY from EECs through activating FFA2 or FFA3 [145,279]. EECs (L-type) that have FFA2 or FFA3 receptors relay signals either to the ENS or the CNS through related extrinsic neurons. The fact that FFA3 receptors were also identified in the peripheral nervous system shows that SCFAs are important top-down signaling molecules in the GBA [280]. Nevertheless, the level of SCFAs is low in those with anxiety or depression [281].

The gut-microbial production of acetate stimulates the secretion of ghrelin that crosses the BBB, activating the cerebral production of GABA [282,283,284]. It intervenes in self-control and emotion regulation as well as in the learning process [285]. However, acetate also has pro-inflammatory effects [286]. Conversely, propionate, as a gut-microbial metabolite, has neuroprotective effects by preventing a decrease in NPY; however, this is associated with weight gain [271]. Butyrate has antidepressant effects, intervening in social dominance behavior [271]. In preclinical models of Alzheimer’s disease, butyrate positively affected pathology and memory performance [287]. The fixation of butyrate on FFA3 receptors in colonic myenteric neurons leads to an overexpression of acetylcholine transferase (studies on rats) [262].

Bile acids, considered bioactive signaling molecules, reflect another complex and dynamic association between the gut microbiota and metabolites produced by various species. The receptors for bile acids are identified at both the gut level and within the CNS [288,289]. The majority of primary BAs are absorbed in enterohepatic circulation, with only a small proportion of BAs reaching the colon. They attach to receptors like farnesoid X receptor (FXR) and TRG5 [290]. TRG5 receptors regulate food intake (determining satiety) [291]. Although BAs do not cross the BBB, with a few exceptions for UDCA and tauro-UDCA, the FXR receptors are present in brain neurons, microglia, and astrocytes [292,293]. Under normal conditions, the indirect effect of BAs prevails through their action on ECs, promoting the release of Ser, as well as on many subtypes of EECs, such as those that release GLP-1 and PYY [290].

The farnesoid X receptor (FXR) is a nuclear receptor that binds to bile acids. Additionally, vitamin D and pregnane X receptors are also nuclear receptors that facilitate bile acid coupling. On the surface of epithelial cells, there is Takeda G-protein receptor 5 (TGR5), also referred to as G protein-coupled bile acid receptor 1 (GPBAR1) [294].

Another contribution of microbiota to GBA is represented by its capacity to modify various bioactive molecules originating from ingested food.

For instance, indole, resulting from Trp, modifies GLP-1 secretion in different directions in acute situations (increase in secretion) and in the long term (decrease in secretion) [99]. The intra-cecal administration of indole determines the activation of the related vagal fibers [295].

Lipopolysaccharide and hydrogen sulfide, both microbiota products, influence GBA. LPS negatively affects epithelial barrier integrity [296], and hydrogen sulfide seems to induce GLP-1 secretion. However, the effects of hydrogen sulfide on GLP-1 release are controversial [297,298].

## 7. miRNAs’ Roles

Recent studies have revealed the significance of microRNAs (miRNAs) in intestinal homeostasis and the bidirectional interaction between these small, non-coding molecules and the gut microbiota [299]. The most important role of miRNAs is considered to be the post-transcriptional regulation of gene expression through binding to specific sites on messenger RNA (mRNA) [299]. miRNAs are involved in processes related to cellular growth, differentiation, and apoptosis [300].

Some authors have proposed miRNAs as key components in GBA communication [301]. Various experiments on germ-free mice, followed by recolonization, have shown the roles of miRNAs in critical brain regions. For example, ablation of a crucial enzyme (DICER) which is involved in miRNA processing leads to anxiety in mice [302]. Foster et al. demonstrated that the microbiome can regulate stress and anxiety through miRNAs in mice [127]. Similar results were also reported by Li Q et al. (2023), as they found that increased miR-206-3p levels in rodent subjects were associated with increased anxiety via activation of the Cited2/STK39 genes [303]. At the same time, other authors have shown the role of miRNAs in memory formation and even sociability [304,305]. However, the implications in human subjects have yet to be fully uncovered and remain an important area of ongoing research.

Host miRNAs are considered a regulatory mechanism through which the host maintains equilibrium within the microbiota and prevents dysbiosis [306]. The miRNAs produced by intestinal epithelial cells and Hopx cells are taken up by bacteria in the intestinal lumen, affecting bacterial gene expression and microbiome composition by targeting bacterial mRNAs [306]. For example, miRNAs may affect the growth or virulence of *Escherichia coli* and *Fusobacterium nucleatum* [306].

To date, it is well established that dysbiosis, characterized by an overgrowth of specific microbial species, further disrupts host microRNA (miRNA) expression, thereby exacerbating the activation of inflammatory pathways. miRNAs have been shown to play a critical role in the regulation of intestinal barrier permeability, modulation of key inflammatory networks, such as NOD2 and IL-23 signaling networks, as well as the alteration of immune responses and the activation of autophagy mechanisms [181]. Furthermore, the reverse is true; the gut microbiota influences host miRNA expression [307]. The gut microbiota modulates the human host’s miRNA production through microbial components (such as LPSs) or their metabolites, like SCFAs [307]. In this way, bacterial metabolites intervene in inflammation regulation, host metabolic processes, and even proliferation (sometimes involved in carcinogenesis) [307]. For example, SCFAs can lead to the expression of anti-inflammatory miRNAs, protecting against inflammatory diseases [308].

## 8. Methodological Limitations

There are some key methodological limitations that frequently occur in studies examining the relationship between the gut microbiota and the CNS. These limitations are more obvious when transitioning from animal to human models.

### 8.1. Differences Between Animal and Human Models

While rodent models allow mechanistic insights (like those studied in germ-free mice), they may fail to reflect the complexity of human physiology and especially psychology. One important aspect is that the animal gut microbiota differs markedly in diversity and abundance from humans, and germ-free animals have underdeveloped immune and nervous systems, which may exaggerate the effects of microbiota interventions. Moreover, animal behaviors do not model human emotional or cognitive states [309].

### 8.2. Small Cohort Sizes and Lack of Power

Many human studies enroll a small number of participants, limiting statistical robustness. The populations studied were heterogeneous (especially regarding mental health conditions), making it difficult to detect clear microbiota patterns [310].

### 8.3. Variability in Microbiome Sequencing Technologies

The sequencing methods used were inconsistent from one study to another (16S rRNA gene sequencing vs. metagenomic sequencing), and there was variation in the collection, sample handling, and storage methods, which may have affected the microbial profiles [311,312].

### 8.4. CNS Outcome Measurement

Most studies rely on self-reposting scales and lack physiological and imaging correlates. They also often last only a few weeks, while some effects may take longer to appear [313].

### 8.5. Environmental and Lifestyle Confounders

Uncontrolled or poorly monitored diets may influence microbiota independent of the study intervention, while antibiotics and other medications may significantly alter the microbiome. Other CNS-relevant factors, like stress, sleep, and physical activity, are difficult to control and measure [314].

## 9. Future Directions and Outstanding Questions

Although new data is emerging constantly, current findings regarding the relationship between the gut microbiota and the central nervous system are mostly cross-sectional or short-term. Long-term studies are needed to link the gut microbiome dynamics to CNS outcomes. Reproducibility and meta-analyses are hampered by the fact that studies use diverse methods for sampling (faecal kits vs. stool), sequencing, metabolomics, and neuroimaging. The most spectacular data is emerging from animal studies which show the effects of the SCFAs, vagal signaling, immune pathways, and microglia modulation and the fact that the gut microbiota can influence host behavior. While causality is supported in animals, human translation is ongoing, and more mechanics and longitudinal human studies are needed to confirm and refine these possible links [315].

There are a few important questions to be addressed in the future:

- Temporal causality: Do the shifts in the gut microbiome precede, follow, or co-occur with neural or behavioral changes in disorders like depression, autism, and dementia? Can the dysbiosis be reversed in order to improve the outcomes of patients [310]?
- Biomarker validity: Which microbial metabolites or immune markers reliably predict CNS health or disease [316]?
- Clinical translation capacity: Are interventions like fecal microbiota transplants, targeted probiotics, diet, or molecular therapies effective and safe in neuropsychiatric or neurodegenerative disease? Can these interventions improve the well-being of humans [317]?
- Personalized interventions: How does inter-individual variation (baseline microbiome, diet, and genetics) influence the response to psychobiotics or fecal microbiota transplant, and can we tailor therapies accordingly [317]?

## 10. Conclusions

The recent growing evidence emphasizes the gut microbiota’s role in modulating the nervous system through a two-way pathway known as the gut-brain axis. Microbial by-products such as short-chain fatty acids and neurotransmitter-like molecules, along with immune and endocrine signaling, influence neurophysiological functions, mood, cognition, behavior, and autonomic responses. Moreover, nervous pathways—including the parasympathetic nerve and the endocrine stress axis—modulate gut microbiota composition and activity. Dysbiosis is involved in a wide range of behavioral and psychiatric disorders, as well as the modulation of neurological inflammatory response. Better knowledge of these interactions might open up new perspectives regarding therapeutic interventions that target the microbiota.

## Figures and Tables

**Table 1:** Probiotic, prebiotic, or symbiotic effects on gut-brain axis in major depressive disorder and anxiety disorder. (PCR = polymerase chain reaction, IBS = irritable bowel syndrome.)

Authors and Year	Number of Subjects	G-B-M Intervention	Disease	Intervention	Outcomes
Boehme, M. et al. (2023) [237]	47 patients	High doses of probiotic (*Bifidobacterium longum* (BL) NCC3001) and its effect on stress.	Stress	Questionnaires regarding stress and its effects on daily activities. Cortisol levels from saliva. Analysis of fecal abundance of *Bifidobacterium longum* (BL) NCC3001.	Significant perceived stress and improvement in sleep were reported after probiotic supplementation (*p* = 0.017 and *p* = 0.037). Acute stress response represented by the salivary cortisol levels was decreased by probiotic administration. Probiotic supplementation decreased the overall stress- and anxiety-related symptoms.
Önning, G. et al. (2023) [238]	132 patients	*Lactiplantibacillus plantarum* HEAL9 can ameliorate cognitive functions in stress-related disorders.	Stress	Questionnaires regarding stress, mood, and quality of life. Cortisol serum levels as well as serum levels of ransforming growth factor β 1 (TGF-β1), galectin-3, fractalkine/CX3CL/CX3CL1, brain-derived neurotrophic factor (BDNF), tryptophan, L-kynurenine, and high-sensitivity C-reactive protein (hs-CRP).	Probiotic supplementation was associated with improvement in stress levels. (Cortisol levels were significantly reduced; *p* < 0.039). Improvement in sleep quality was observed after 12 weeks of trials. Significant improvement in short-term memory was observed (*p* = 0.003). No significant changes were reported among biomarker levels.
Casertano, M. et al. (2024) [239]	44 patients	Probiotic supplement consisting of Levi-lactobacillus brevis P30021 and Lactiplantibacillus plantarum P30025 ant its ability of having a positive impact on mental well being.	Stress	Genomic analyzation of gut microbiota Cognitive and stress levels assessment Neurotransmitters analyzation	No effect on stress levels were observed, however depressive symptoms were ameliorated (*p* = 0.034). No significant changes in gut microbiota were reported, as well as no correlation of neurotransmitters weas reported.
Martin, F.P. et al. (2024) [240]	36 patients	*Bifidobacterium longum* (BL) NCC3001 decreases emotional reactivity and ameliorates depression via modulation of gut microbiota.	IBS	PCR analysis of BLNCC3001. Brain mapping activity.	Higher levels of butyric acid were associated with improvement in clinical symptoms related to depression, as well a decrease in activation of the amygdala. The abundance of BLNCC3001 ameliorated anxiety and depressive symptoms via increasing the synthesis of butyric acid.
Sarkawi, M. et al. (2024) [241]	110 patients	Effects of high doses of *Lactobacillus acidophilus* LA-5 and *Lactobacillus paracasei* L. CASEI-01 on IBS symptoms.	IBS	Questionnaires regarding IBS symptoms and quality of life. Serum levels of serotonin and cortisol.	Significant improvement in IBS-related symptoms after probiotic supplementation (*p* < 0.05). Significant increase (*p* = 0.002) in serotonin levels after probiotic treatment but no change in cortisol levels.
Chao, W.-C. et al. (2024) [242]	31 patients	Yoga and probiotic supplementation	IBS	Genomic analyzation of gut microbiota. Fitness and quality of life assessment.	Significant changes in gut microbiota were observed, especially in *Klebsiella* and *Prevotella* species (*p* < 0.05). Improvements in fitness levels were also observed.

**Table 2:** Microbial metabolites and nervous system effects.

Microbial Metabolite	Produced by	Mechanism of Action	Effects on the CNS
Short-chain fatty acids [214]	Firmicutes (*Lactobacillaceae, Ruminococcaceae, Lachnospiraceae)* *Bifidobacteriaceae*	Influence microglial function Influence gene expression via HDAC inhibition	Anti-inflammatory role Enhance blood–brain barrier integrity Influence mood and cognition
Tryptophan metabolites [263]	*Lactobacillus* *Bifidobacterium*	Influence serotonin synthesis	Regulate mood, anxiety, and cognitive function
Gamma-aminobutyric acid (GABA) [264]	*Lactobacillus* *Bifidobacterium*	Modulates neuronal excitability	Regulates anxiety and may have antidepressant properties
Dopamine and precursors [265]	*Bacillus* spp. *Escherichia* spp.	Influence host dopamine pathways	May affect motivation, reward, and motor control
Histamine [266,267]	*Lactobacillus reuteri*	Modulates immune responses Acts via H1 and H2 receptors in CNS	Can influence wakefulness, appetite, and cognitive processes
Lipopolysaccharide [268]	*Gram-negative bacteria*	Activates systemic inflammation via TLR4 signaling	Neuroinflammation Role in depression and cognitive decline
Peptidoglycans [269]	*Gram-positive bacteria*	Activate innate immune responses	May have a role in neuroimmune interactions
Phenylacetylglutamine [270]	*Christensenellaceae*, *Ruminococcaceae*, *Lachnospiraceae*, *Bacteroidetes*, *Firmicutes*, *Proteobacteria*, some Gram-negative bacteria	Found in CNS but mechanism not clear	Linked to cardiovascular and possibly cognitive functions
**SCFA**	**Produced by**	**Mechanism of Action**	**Effect on CNS**
Acetate [271]	Bacteroides Prevotella Firmicutes	Activates hypothalamic neurons and modulates glial activity	Influences appetite and energy balance Possible neuroprotective effects
Propionate [271]	Bacteroidetes Firmicutes	Influences neurotransmission Interacts with G-protein-coupled receptors (GPCRs)	Anti-inflammatory effects May improve memory Potential anxiolytic effects
Butyrate [271]	*Clostridium* spp., *Eubacterium*, *Roseburia*, and *Blautia* *Faecalibacterium prausnitzii*	Histone deacetylase (HDAC) inhibitor (role in gene expression) Modulates microglia and enhances blood–brain barrier	Anti-inflammatory effects Improves neuroplasticity Reduces anxiety and depression
Isobutyrate [272]	Protein fermentation bacteria: *Clostridium* spp., *Desulforhabdus amnigenus*	Less studied, but may influence signaling through GPCR pathways	Not clear
Valerate [273]	Fermentation of amino acids: *Clostridium* spp.	HDAC inhibitor	May have anti-inflammatory role May have neuroprotective properties
Isovalerate [274]	Protein and amino acid fermentation: Bacteroides and *Clostridium* spp.	May affect microglial activation	May have a role in neuroinflammation

## Data Availability

Not applicable.
